# Thermogenic adipose tissue in energy regulation and metabolic health

**DOI:** 10.3389/fendo.2023.1150059

**Published:** 2023-03-20

**Authors:** Siwen Xue, Derek Lee, Daniel C. Berry

**Affiliations:** Division of Nutritional Sciences, Cornell University, Ithaca, NY, United States

**Keywords:** beige adipocyte, brown adipocyte development, aging, obesity, metabolism & endocrinology

## Abstract

The ability to generate thermogenic fat could be a targeted therapy to thwart obesity and improve metabolic health. Brown and beige adipocytes are two types of thermogenic fat cells that regulate energy balance. Both adipocytes share common morphological, biochemical, and thermogenic properties. Yet, recent evidence suggests unique features exist between brown and beige adipocytes, such as their cellular origin and thermogenic regulatory processes. Beige adipocytes also appear highly plastic, responding to environmental stimuli and interconverting between beige and white adipocyte states. Additionally, beige adipocytes appear to be metabolically heterogenic and have substrate specificity. Nevertheless, obese and aged individuals cannot develop beige adipocytes in response to thermogenic fat-inducers, creating a key clinical hurdle to their therapeutic promise. Thus, elucidating the underlying developmental, molecular, and functional mechanisms that govern thermogenic fat cells will improve our understanding of systemic energy regulation and strive for new targeted therapies to generate thermogenic fat. This review will examine the recent advances in thermogenic fat biogenesis, molecular regulation, and the potential mechanisms for their failure.

## Introduction

Mammalian survival depends upon metabolic plasticity by responding to variations in food availability and environmental signals (1). These dynamic changes often trigger cellular adaptation, metabolic reprogramming, and altered energy homeostasis. For example, white adipose tissue can rapidly expand in response to a positive energy balance (2). This expansion relies on storing excess nutrients as triglycerides within lipid droplets of existing white adipocytes (hypertrophy). Alternatively, new white adipocytes can be recruited from adipocyte progenitor cell (APC) pools located within adipose depots (hyperplasia) (3). Opposing white adipose tissue accumulation and expansion are thermogenic fat cells—brown and beige adipocytes (4). Thermogenic fat cells can be recruited and activated in response to sympathetic nervous system (SNS) activation, such as cold temperatures (5, 6). Once activated, these cells utilize and combust glucose and free fatty acids to drive thermogenesis rather than cellular energy production (7). These observations highlight the importance of adipose tissue as a highly dynamic organ responding to nutritional cues and environmental stimuli to coordinate systemic metabolism. Thus, caloric excess and reduced variation in temperature challenges could support uncontrolled expansion and accumulation of white adipose tissue, potentiating metabolic dysregulation.

**Figure 1:**
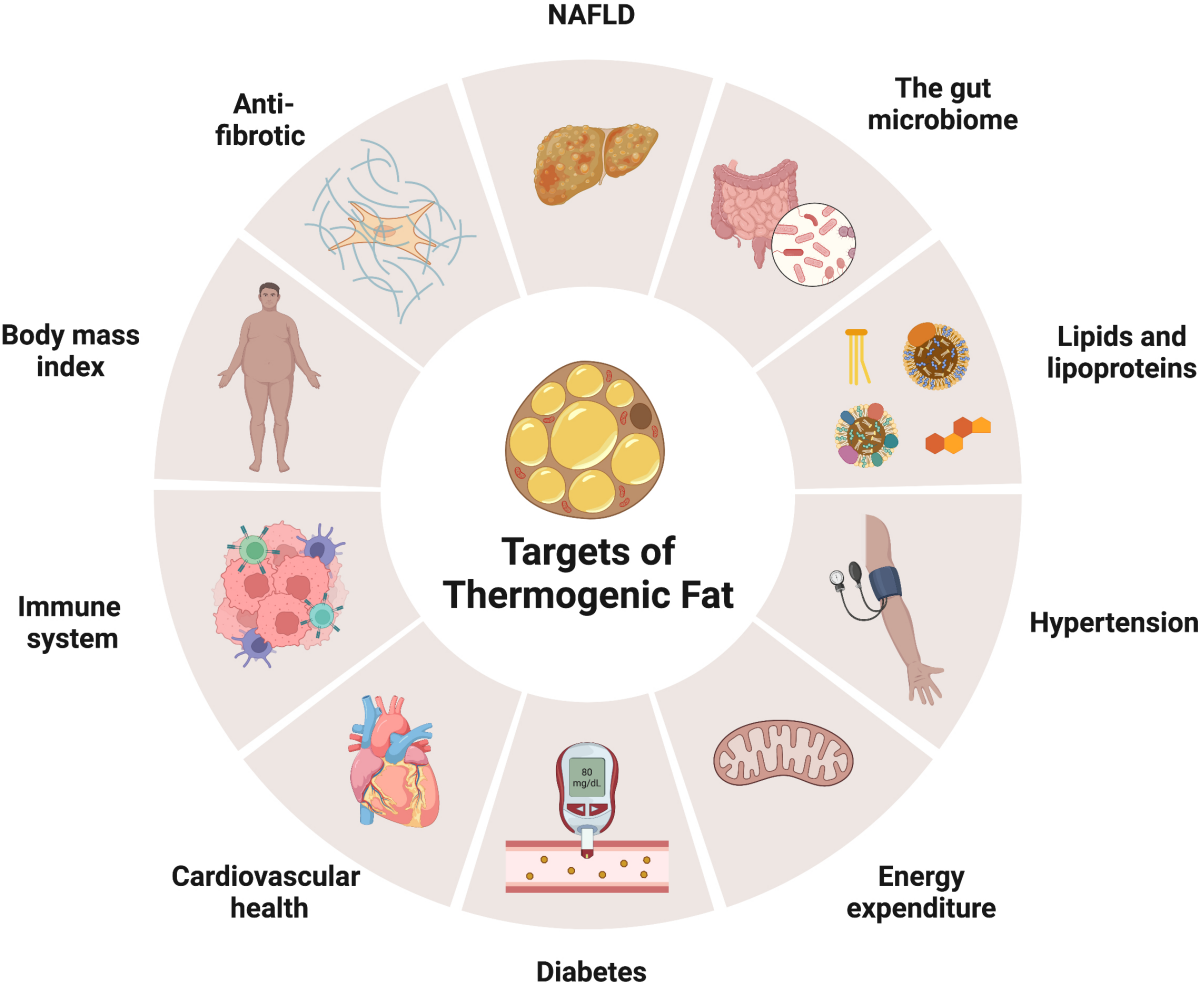
Metabolic hallmarks of thermogenic fat. Chronic overnutrition promotes adipose tissue malfunction, increasing the risk for developing metabolic diseases, such as type II diabetes, hypertension, fatty liver disease, and cardiometabolic disease, and premature death. Thermogenic fat (brown and beige) biogenesis and activation offers promising potential to improve systemic energy expenditure and ameliorate obesity and its associated comorbidities. Illustration created with BioRender.com.

Indeed, the maladaptation of adipose tissue metabolic flexibility is strongly associated with obesity and chronic metabolic disease (8). For instance, sustained obesogenic signals foster white adipose tissue dysfunction, increasing the risk of developing type 2 diabetes, arteriosclerosis, hypertension, dyslipidemia, fatty liver disease, and premature death (9). Further, chronic overnutrition also augments adipose tissue inflammation, which modifies adipocyte function and insulin responses and, consequently, leads to adipocyte cell death (10). Adipose tissue dysregulation originates from various changes in mitochondria function and biogenesis, extracellular matrix accumulation, adipokine secretion profiles, lipid composition, and thermogenic fat cell development. In particular, the inability to generate thermogenic fat cells in aged and obese humans represents a significant clinical challenge to counteract white adipose tissue accumulation and expansion (11). Thus, strategies devised at increasing or rejuvenating thermogenic fat biogenesis and activation could be complementary treatments for obesity and its associated metabolic diseases (**Figure 1**). Here, we review the current understanding of thermogenic fat formation and decline and its benefit and discuss strategies to restore thermogenic fat in aged and obese humans.

## Brown adipose tissue

Three types of mammalian adipocytes exist—white, beige, and brown—spatiotemporally organized throughout the body (12–14). White adipocytes are specialized for energy storage but regulate various systemic physiologic and metabolic responses such as appetite, reproduction, and glucose and lipid metabolism (15). In contrast to the energy storage function of white adipocytes, brown and beige fat cells perform thermogenesis to increase energy expenditure. In 1551, Conrad Gesner first described and harbingered brown adipose tissue (BAT) in marmots as a mammalian hibernating gland (16). Yet, somewhat surprisingly, its function remained a mystery for over four centuries. In the early 1900s, preliminary studies used *in vitro* culturing and organotypic slices to reveal that BAT respired more than white fat or other non-adipose organs (16–18). Strikingly, the effect on respiration was heightened in response to hibernation and cold exposure, suggesting that BAT is a metabolically active organ required for temperature defense (19). Indeed, cold-adapted mammals utilize non-shivering thermogenesis to achieve temperature defense and stimulate BAT remodeling by increasing sympathetic innervation and vascularization (16). For instance, Hausberger and Widelitz showed that the vascularity of BAT within rats was four to six times higher than white adipose tissue but appeared comparable to resting skeletal muscle (20). The abundance of vascularity coupled with the thermogenic action of BAT provided an opportunity to establish the biothermic flow of blood and the vascular configuration within BAT lobes (16). These formative studies on the BAT vascular structure revealed the thermal conduction, exchanges, and organ series required for BAT to maintain homeothermy.

Differentiated brown adipocytes reside within BAT depots that are visually distinct compared to white fat cells. For instance, white adipocytes are circular, lack cytoplasmic volume, and contain a single large lipid inclusion (21). Conversely, brown adipocytes appear smaller, are polygonal, have more cytoplasmic volume, and have a dispersion of many tiny lipid droplets (multilocular) (21, 22). The multilocular lipid droplet organization of brown adipocytes is thought to assist in improving thermogenic substrate utilization by enhancing the lipid droplet surface area-to-volume ratio (23). Lipid droplet-associated proteins are thought to inhibit droplet coalescence, maintaining the multilocular phenotype of brown adipocytes (24). A recent study by Tontonoz and colleagues revealed that calsyntenin-3β (CLSTN3β)—a member of a family of proteins that regulate intracellular trafficking, synaptic function, and neuronal communication (25)—acts to confine lipid droplet-associated proteins averting droplet fusion to maintain the multilocular lipid droplet morphology (26).

Brown adipocytes contain numerous mitochondria that are large, closely packed, and contain laminar cristae compared to white adipocyte mitochondria, which appear small, elongated, and contain randomly oriented cristae (16). Differences in mitochondria number and structure correspond to the functional roles between energy utilization versus storage. In agreement, brown adipocytes express restricted transcription factors and co-factors that cooperate to induce mitochondria biogenesis and facilitate fatty acid oxidation. For example, peroxisome proliferator-activated receptor-γ (PPARγ) coactivator-1α (Pgc1α) is a transcriptional co−activator that unequivocally controls mitochondria biogenesis within brown adipocytes (27). While Pgc1α is noncompulsory for BAT development, it is required for cold temperature- and β3-adrenergic receptor (Adrb3)-agonist-induced brown adipocyte activation and thermogenesis. Consistent with this notion, Pgc1α is highly induced in response to cold exposure, allowing Pgc1α to interact with various transcriptional regulators (28). Thus, the discovery of BAT and brown adipocytes signified a mammalian adaptation designed to protect homeothermy.

## Thermogenic activation

BAT relies on a dense network of sympathetic neurons innervating the tissue to activate thermogenesis. Indeed, denervation studies have demonstrated the importance of sympathetic neurite dispersion throughout BAT to activate thermogenesis (5, 29, 30). To engage the SNS, thermoreceptors within the skin detect subtle cooling in the ambient temperature, which transmits these sensations along afferent neurons to the hypothalamus (31–33). The hypothalamus engages the postganglionic sympathetic nerve termini to release catecholamines (norepinephrine) into the BAT milieu to activate Adrb3 on brown adipocytes. Specifically, Adrb3 is a G protein-coupled receptor that engages adenylate cyclase activation in response to norepinephrine binding (34). Unlike other Adrbs, Adrb3 is primarily expressed in adipose tissue to initiate lipolysis and thermogenesis (35). Upon norepinephrine binding, Adrb3 is stimulated, initiating the downstream phosphorylation and activation of protein kinase A (PKA) and cyclic AMP (cAMP), facilitating lipolysis, beta-oxidation, and the transcriptional upregulation of thermogenic and mitochondrial biogenic proteins (36–38). Catecholamine release also stimulates brown adipocyte progenitors within the BAT stromal vascular fraction (SVF) to differentiate (29, 39–41). Moreover, brown fat sympathetic innervation may also serve as an interorgan communication network, specifically for those tissues with diffuse sympathetic innervation, such as white fat (42–44). Interestingly, the cholinergic receptor (CHRNA2), a nicotinic acetylcholine receptor, has also been shown to stimulate thermogenic fat development by activating PKA and cAMP signaling, independent of Adrb3 activation (45). What’s more is that immune cells located within subcutaneous fat appear to produce acetylcholine potentiating CHRNA2 activation (46). Regardless of activation, cAMP signaling culminates in the transcriptional upregulation of uncoupling protein 1 (Ucp1). Ucp1 is a mitochondrial inner membrane protein that reduces the proton potential between the mitochondrial intermembrane space and matrix, collapsing the electron transport chain and preventing ATP production (47). The resulting changes in the ATP/ADP ratio elevate the rate of substrate oxidization, such as lipids, continuing the uncoupling process of the electron transport change and the generation of heat (48).

While Ucp1 activity and regulation have been well-characterized in energy uncoupling, several studies have revisited the potential for Ucp1-independent thermogenic mechanisms. These studies originated from observations related to Ucp1 genetic modeling and the low expression level of Ucp1 in beige fat relative to brown adipocytes (49, 50). Consistent with Ucp1-independent thermogenic pathways, Ucp1-null mice show varied effects in response to cold exposure and diet-induced obesity, which appear to relate to vivarium temperature conditions and genetics (51). Yet, adipocyte-specific overexpression of Ucp1 prevents genetic obesity (52). Nevertheless, emerging adipocyte metabolic studies have focused on cellular redox status, purine nucleotide pool size, calcium signaling, succinate bioavailability, and creatine cycling as Ucp1-dependent and independent thermogenic mediators (reviewed in (53, 54)). Modulating several of these pathways in Ucp1-deficient mice, has revealed adaptations in macronutrient utilization and protection from diet-induced obesity, showcasing Ucp1-independent thermogenic pathways (54). Adding further to this notion is the identification that brown adipocytes appear to differ in Ucp1 status (low vs high) (55). Using the AdipoChaser mouse models combined with single-cell RNA sequencing and 3D tissue profiling, Song et al. (2020) found a coexistence of two brown adipocyte subpopulations—brown adipocytes that highly express Ucp1 and brown adipocytes that lowly express Ucp1. For instance, low Ucp1 expressing brown adipocytes were associated with lower adiponectin gene expression, larger lipid droplets, and lower mitochondria content (55). Even though Ucp1 expression is lower, these brown adipocytes appear to be specialized in fatty acid uptake. Yet, upon cold stimulus, low Ucp1 expressing brown adipocytes interconverted into high Ucp1 expressing brown adipocytes and vice versa in response to thermoneutral temperatures (55). These findings indicate that brown adipocytes are highly heterogenetic tissue and may be specialized in metabolic responses. Further, these findings suggest that some thermogenic fat cells are poised to become activated or deactivated in response to temperature changes. Overall, thermogenic fat cells appear highly dynamic and utilize various substrates to modulate energy uncoupling and function in Ucp1- dependent and independent mechanisms. The potential employment of these pathways may reflect substrate bioavailability, Ucp1 post-translational modification, and thermogenic fat stimuli (56). Moreover, the biological significance of Ucp1-independent thermogenic pathways has yet to be demonstrated in humans (57).

## Cellular ontology of brown adipocytes

**Figure 2:**
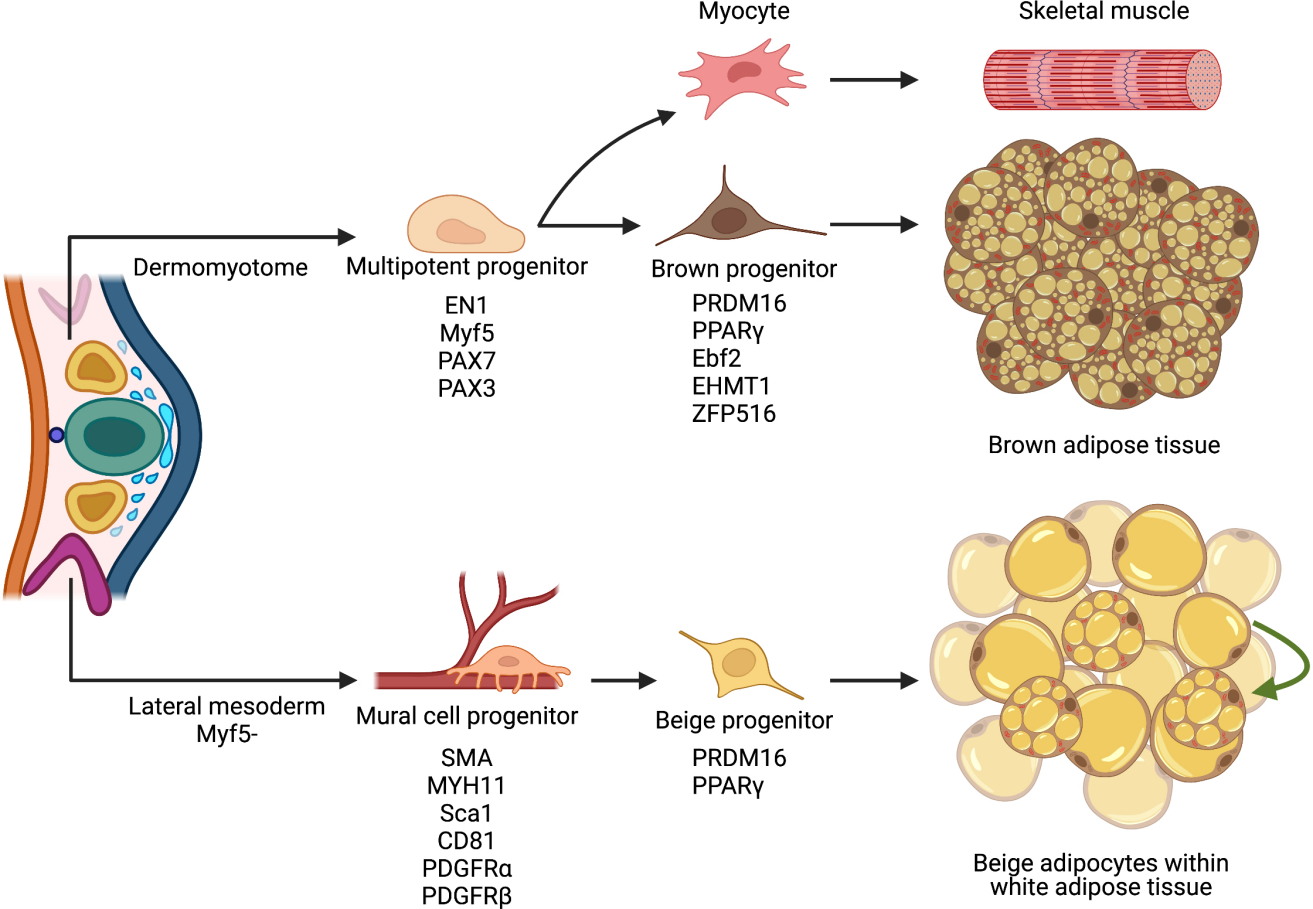
Cellular ontology of thermogenic fat biogenesis. Multipotent progenitor cells located within the paraxial mesoderm of the dermomyotome can give rise to brown fat precursors that express myogenic markers. Though brown fat and skeletal muscle may share similar cellular ancestry, specific transcriptional factors regulate and facilitate brown adipocyte lineage specification and determination. Contrastingly, white and beige adipocytes arise from a Myf5- mesodermal origin, resemble mural cells, and reside in a perivascular niche. Upon cold exposure, these cells invoke a thermogenic transcriptional program and differentiate into beige adipocytes. Illustration created with BioRender.com.

White and brown adipose depots are speculated to develop from a mesodermal cellular lineage, sharing a commonality with skeletal muscle, bone, and connective tissue (58). Like other mesodermal tissues, brown adipocytes are specified during embryogenesis and develop into distinct depots throughout the body. Indeed, mouse interscapular brown fat can be histologically observed as early as embryonic day 15.5 (E15.5) (59, 60). Yet, advances in cellular lineage tracing using the adiponectin promoter (AdipoChaser (61)) suggest that brown adipocytes can develop as early as E10 (55). These primitive BAT depots seem to be generated by hyperproliferative fibroblast-like cells that remain lipid depleted until the peripartum period (62, 63). To gain insight into the cellular ontology, early fate-mapping studies of embryonic precursors showed that brown adipocytes derived from the paraxial mesoderm of early somites within the dermomyotome (**Figure 2**). For example, using a marker of the central dermomyotome, engrail-1—a transcription factor involved in embryonic development and patterning (64, 65)—revealed that brown fat and skeletal muscle shared a common cellular history (66). In agreement, myogenic factor five (Myf5), an early myogenic transcription factor, lineage reporting studies demonstrated the shared cellular lineage between skeletal muscle and brown fat (67–69). Consistently utilizing two skeletal muscle transcription factor driven genetic tools, Pax3 and Pax7 (70, 71), to mark the developing muscle lineage, again showed embryonic muscle progenitors’ ability to develop into brown fat cells (71). While developmental muscle progenitors appear to be multipotent, myoblasts, a muscle lineage-committed progenitor, cannot generate brown adipocytes, suggesting lineage restriction after skeletal muscle commitment (72–74). Overall, genetic tools marking early embryonic muscle progenitors show the capacity to generate skeletal muscle and brown adipocytes.

Broadly, the growing lineage tracing evidence suggested that brown and white adipocytes arise from distinct embryonic progenitors. However, the recent re-examination of the Myf5 lineage identified that specific white adipocytes within restricted adipose depots are generated from a myogenic lineage (68, 69). Thus, revealing the mosaic contribution of the Myf5 lineage to white fat development. Consistent with this notion, Rodeheffer and colleagues, using the somite-specific Meox-1-Cre, showed that both dorso-axial white and brown adipocytes derive from the somitic mesoderm (75). In contrast to previous lineage data, only a subset of interscapular brown and white adipocytes originate from a Pax7+ lineage. These data favor an epaxial, not central, dermomyotome lineage for interscapular fat and the selective co-development of white and brown adipocyte depots (75). However, differences in lineage analysis studies could be attributed to Cre driver development (transgenic or knockin), the mouse strain genetic background, sex, reporter recombination efficiency, and spontaneous recombination and marking, which could influence cellular contribution and specification (76–79).

Because skeletal muscle and brown adipocytes share a common developmental cellular origin, decisive transcriptional machinery must influence cellular fate. Indeed, PR domain zinc-finger protein 16 (PRDM16) acts as a transcription factor molecular controller, favoring genes involved in brown fat cell formation over myogenesis (67). Even though Prdm16 is not required for brown adipocyte embryonic development, modulating Prdm16 can influence cell fate. For instance, *in vitro* overexpression of Prdm16 in a myoblast cell line—C2C12—induced brown adipocyte differentiation and induction of a thermogenic program. Conversely, downregulating Prdm16 in brown preadipocytes stimulates myotube formation (67). While Prdm16 is important in controlling brown adipocyte identity, the regulation and protein stability of Prdm16 has been undefine (80). Recent inroads into Prdm16 protein stability revealed a novel ubiquitin E3 ligase complex that catalyzes the polyubiquitination and degradation of the Prdm16 protein. Metabolically, when Prdm16 protein stability was extended by ablating the ubiquitin E3 ligase complex, mice resisted diet-induced obesity (81).

In addition to Prdm16, Early B Cell Factor-2 (Ebf2), an early developmental transcription factor, has been shown to specifically mark embryonic brown fat progenitors (63). In agreement, Ebf2 expression seems restricted to the brown fat somitic mesoderm lineage and is undetected within other lineages, including muscle. Consistent with this notion that Ebf2 is an initial driver of brown adipocyte lineage commitment and Ebf2 can cooperate with chromatin remodeling machinery to alter transcription factor accessibility at adipogenic genes such as the nuclear hormone receptor, peroxisome proliferator activated receptor gamma (Pparγ) (82). Notably, Pparγ is necessary and sufficient for adipogenesis and adipocyte regulatory function (83–86). Further studies examining inductive cues initiating Ebf2 expression and adipogenic commitment will provide insight into cellular fate and brown adipocyte lineage commitment. Beyond Prdm16 and Efb2, several other factors such as BMP7, C/EBPβ, EHMT1, EWS, and ZFP516 have been shown to facilitate brown fat adipocyte lineage specification and determination (87–90). These studies suggest multiple factors act as lineage switches and rheostats between skeletal muscle and brown fat cell types (**Figure 2**). In addition, these factors also appear to control brown adipocyte identity and thermogenic function.

While the developmental lineage progression of BAT has been recognized, less is known about adult BAT progenitor identity, induction, and regulation. Cold stimulus and diet are strong regulators of adult BAT homeostasis and growth. For example, thermoneutral conditions, an ambient temperature where the heat produced and heat loss are at equilibrium, diminish sympathetic tone, decreasing the requirement of the thermogenic program (50, 91). A reduction in thermogenic cues also supports brown adipocyte lipid accumulation and the acquisition of a unilocular white adipocyte-like appearance—hypertrophy. In contrast, cold exposure increases BAT mass *via* new brown adipocyte recruitment from a progenitor pool—hyperplasia. What might comprise the brown adipocyte progenitor pool? Genetic fate mapping, using platelet-derived growth factor receptor alpha (Pdgfrα) driven inducible Cre-reporter system, established that perivascular adventitial cells could generate brown fat cells (92). Notably, newly generated brown adipocytes from Pdgfrα+ cells predominantly occurred along the BAT dorsal edge. Additionally, Pdgfrα+ cells had the propensity to divide prior to adipogenesis (92). However, a potential confound is that Pdgfrα, a receptor tyrosine kinase involved initiating cellular signaling cascades that regulate cell growth and differentiation, is involved in the development and maintenance of numerous tissues, including the vascular system, the nervous system, organs, and connective tissues (93–96). In addition to Pdgfrα+ cells, single-cell RNA sequencing (scRNA-seq) technology identified a population of vascular smooth muscle cells expressing the transient receptor potential cation channel subfamily V member 1 (TRPV1), a membrane-bound calcium permeable channel that is part of the somatosensory system, which can be activated by temperature and pungent compounds (97, 98). Yet, overall there appears to be a lack of information on the adult brown adipocyte lineage and further investigation into the implications of the presumptive adult brown adipocyte precursor cell and their utility awaits.

## Identification and cellular ontology of beige adipocytes

In the early 1980s, researchers were cold acclimating female mice, they observed patches of brown-like adipocytes within perigonadal depots of white adipose tissues (14). Subsequently, similar studies using the selective Adrb3 agonist, CL316,243, revealed the occurrence of brown-like adipocytes within white adipose tissues, underscoring the SNS as a regulator of this “browning” process (99). Initially, these mitochondria-rich brown-like cells were thought to be dormant brown adipocytes but were activated by cold temperatures and norepinephrine signaling. However, the next four decades of research revealed a distinctive third type of adipocyte, dubbed beige/brite adipocyte (brown in white adipose tissue). Specifically, beige adipocytes are mitochondria enriched, multilocular in appearance, Ucp1 positive, and thermogenically competent. Initial studies using morphological characteristics and electron microscopy concluded that beige adipocytes derive from the trans-differentiation of mature white adipocytes. In support, Ucp1+ beige adipocyte fate mapping studies showed that thermogenic adipocytes do not vanish after cold temperature elimination; instead, they masquerade as white adipocytes (100). Interestingly, these cells retain a beige fat cell epigenetic memory and await cold re-exposure (101). Adding further to the fray is the possibility that mature Ucp1+ beige adipocytes can self-renewal and propagate in response to Adrb3 agonist administration. Additionally, uncoupling the cell cycle by p16^Ink4a^ deletion facilitated the expansion of beige fat cells independent of stimuli, suggesting that beige adipocytes can perdure in the absence of stimuli and interconversion can be blocked (102). However, the utility of these cells and their contribution to metabolic homeostasis have not been fully elucidated. While these studies suggest that beige fat cells can interconvert from white adipocytes, several interesting questions remain. For instance, do beige-white adipocytes continue to confer metabolic benefits or the number of interconversions and the ability of cells to retain the beige epigenetic memory?

### A mural progenitor for beige adipocytes

Understanding the cellular origins of a given tissue is a complex endeavor but delivers novel insight into tissue regulation and regenerative potential (58, 103). In opposition to the trans-differentiation hypothesis, an adipocyte-specific cell marking study using AdipoChaser suggested that the majority of beige adipocytes were generated from a non-adipocyte cellular pool (61). A follow-up study showed that initial cold exposure requires *de novo* beige adipogenesis. However, interconverting adipocytes were the primary source for the second bout of cold exposure (104). Lineage research then shifted from pre-existing adipocytes to test whether white adipocyte precursors could serve as white and beige fat cell progenitors. For instance, Pdgfrα+ adventitia fibroblasts appear bipotential, emerging into white and beige adipocytes, depending on the stimuli (105). Regarding beige adipogenic potential, Pdgfrα+ cells may only be engaged in response to the Adrb3 specific agonist, CL316,243, and not cold exposure (92, 96, 106). However, the cold temperature results seem mixed with some reports showing a 1-2% contribution to upwards of 20-30% new beige adipocytes emanating from Pdgfrα+ cells (96, 107). These variations in lineage contribution could be related to vivarium temperature conditions, different Cre-driven-reporter recombination efficiency, and the genetic background of the mice. On the other hand, using selective genetic lineage tools against smooth muscle cell markers such as smooth muscle actin (Sma; Acta2), myosin heavy chain 11 (Myh11), and Pdgfrβ, show that a majority of cold-induced beige adipocytes derived from a perivascular cellular source (106, 108, 109). Sma genetic fate mapping assessments demonstrate that Sma+ perivascular cells can generate ~50% of new beige adipocytes after one week of cold exposure (106). In contrast, smooth muscle cells marked by Myh11 and Pdgfrβ only generate beige adipocytes after two weeks of cold exposure (108, 109). Still, the extensive detail of when and where smooth muscle cells form beige fat across white adipose depots remains to be entirely determined. Further, single-cell RNA sequencing identified a CD81 marked perivascular cell population that could generate Ucp1+ beige adipocytes (110). Additionally, CD81+ cells also express Sma and other beige APC markers suggesting these cells may be *bona fide* beige progenitors. Beyond beige progenitor marking, CD81 also appears to control integrin-FAK signaling to initiate beige APC proliferation (110). In addition to mural progenitors, and reasonably so, several studies evaluated if beige adipocytes develop from the dermomyotome lineage, but these studies did not appear to overly support a myogenic developmental lineage (69, 106). However, recent evidence shows that some beige adipocytes are generated from a myogenic-MyoD+ precursor within white adipose tissues (111). Once generated, these MyoD-lineage-positive beige fat cells can catabolize glucose rather than free fatty acids (111). Collectively, multiple cellular pools can be employed to generate beige adipocytes at discrete anatomical locations within the adipose depots. Nevertheless, our understanding of why and when various cell types contribute to beige adipogenesis is underappreciated. Moreover, fate mapping data suggest that white and beige adipocytes may emanate from similar cellular APC pools (85, 106); however, are these pools discrete or overlapping? If overlapping, what might be the difference in cellular signals deciding their fate? The utility of single-cell sequencing coupled with lineage tracing and spatiotemporal niche labeling will help identify potential similarities and differences in progenitor populations and lineage trajectory.

## Differences in beige fat biogenesis

Cold temperatures and Adrb3-agonism utilize aspects of the SNS but may differ in beige fat development and metabolic function. For example, within 24 hours of Adrb3 agonist administration, beige adipocytes can be observed, whereas cold-induced beige fat biogenesis occurs after several days (96). On the other hand, mechanistic studies have implied brown adipocyte activation and beige adipocyte recruitment are similarly activated through *Adrb3*. Yet, *Adrb3* expression appears restricted to mature adipocytes and is undetected within the APC stromal vascular compartment (106, 112). Moreover, Adrb3-induced beige fat development appears to be independent of significant changes in adipocyte proliferation or the amount of DNA content (99, 113). Thus, do Adrb3 agonists stimulate beige APCs to produce beige fat cells? Using fate mapping analysis, neither Sma- nor Myh11-labeled beige APCs generated Adrb3-agonist-induced beige adipocytes (96, 106). For that matter, Pdgfrα+ cells generate roughly ~5-10% of all Ucp1+ Adrb3-agonist-induced beige adipocytes and even less (1-2%) in response to cold temperatures (92, 96, 105). Adipocyte-specific cell marking strategies found that most Adrb3-agonist-induced beige adipocytes emanated from pre-existing adipocytes (96, 104). In further support, Adrb3 genetic necessity tests showed that Adrb3 is dispensable for cold-induced beige fat formation (114). Moreover, mice lacking all three Adrbs (β-less mice) have repressed BAT function and defective beige adipogenic potential (115). Interestingly, if β-less mice slowly acclimate to cold temperatures over a 30-day period, cold tolerance is achieved. These observations suggest the existence of alternative pathways to induce and activate thermogenic fat but may depend on the beiging stimulus.

In contrast to Adrb3, Adrb1, a more ubiquitously expressed adrenergic receptor, is located within the white adipose SVF, not in mature adipocytes (96). Previous reports have posited that Adrb1 may mediate the proliferation and differentiation of classical brown adipocyte progenitors (116). Remarkably, Adrb1 null mice cannot defend their body temperature in response to cold temperature exposure (117). Yet, strikingly, Adrb1 transgenic overexpression invoked robust cold-induced beige fat formation (118). Moreover, treating mice with an Adrb1 inhibitor prevented cold-induced beige fat formation (96). While cold temperatures and Adrb3-agonism result in cAMP signaling, they may act differently on various adipose tissue cells to promote beige fat biogenesis (106). But what might be the advantage of multiple cellular pools for generating beige adipocytes? Do beige adipocytes have different metabolic functions depending on the cellular origin? Future research aimed at teasing apart beige fat development and cellular sources could inform on best practices for inducing beige fat under various conditions such as obesity, aging, or environmental temperatures.

To gain glimpses into metabolic heterogeneity between cold- and Adrb3-induced beige fat biogenesis, Soloway and colleagues used single-nucleus assay for transposase-accessible chromatin sequencing (snATAC-seq) to examine potential differences. Consistently, both stimuli increased accessibility at genes involved in thermogenesis, lipogenesis, and beige adipocyte development; however, the cellular kinetics and magnitudes appeared distinct (119). Moreover, changes in lipogenic genes, for example, were linked to changes in the lipid composition of the adipose tissue. Interestingly, both stimuli decreased the proportion of palmitic acid, a saturated fatty acid, whereas Adrb3 activation favored an increase in monounsaturated fatty acids. In contrast, cold exposure increased the proportion of polyunsaturated fatty acids (119). Consistent with this notion, Adrb3-agonist-induced beige adipocytes express higher levels of medium chain acyl-CoA dehydrogenase (MCAD) protein, an essential mitochondrial enzyme required for lipid oxidation (120). While these findings reveal common and distinct mechanisms of cold and Adrb3-induced beiging, there is a paucity of information regarding the functionality and metabolic consequences of the beige stimuli. For example, might these identified lipids have biological activity that can alter metabolic or thermogenic processes? Indeed, 12,13 diHOME, a fatty acid metabolite, identified as a cold-induced lipokine, can enhance fatty acid utilization and promotes fatty acid uptake and oxidation in myocytes (121). In the same vein, the generation of highly glycolytic beige adipocytes, from MyoD+ APCs, also reveals metabolic substrate specificity (111). Interestingly, the snATAC data (from above) also demonstrated that cold-induced beige adipocytes had increased accessibility at glycolytic processing genes, whereas Adrb3 activation increased cAMP responses. In agreement, glycolytic beige adipocytes are enriched in two enzymes that regulate glycolysis, enolase-1 and the isoenzyme of pyruvate kinase (PKM2), suggesting these cells are specialized in higher glucose utility (111). In addition to increased glucose and free fatty acid consumption, beige adipocytes enhance branch chain amino acid (BCAA) uptake and oxidation (122). This process requires SLC25A44, a BCAA mitochondria transporter. Defects in SLC25A44 impair BCAA transport and oxidation, consequentally, reducing thermogenesis and increasing susceptibility to diet-induced obesity (122). Yet might BCCA uptake and processing differ between beiging stimuli and temperature conditions? Overall, different thermogenic inducers may invoke similar but distinct metabolic programs which may derive from their cellular origin. Together, these studies suggest that thermogenic fat cells have an array of metabolic substrates, but their collective contribution to human thermogenesis and metabolism is unclear.

## Human thermogenic fat

Human brown adipocytes develop in infants and are retained in young children, which is thought to protect newborns from hypothermia (123, 124). But beyond the postnatal years, adult humans were thought to have limited BAT deposits, with only several reports showing detectable human BAT under unique conditions (125). For instance, cold-acclimated outdoor workers in Finland were reported to have BAT deposits located in strategic sites within the body (126). Additional BAT observations were limited to postmortem dissection and autopsies reports (127). In the late 2000s, ^18^F-fluorodeoxyglucose positron emission tomography (FDG/PET) to trace tumor metastasis revealed a compilation of scans showing the appearance of active glucose uptake in human adipose depots (128–131). In some incidences, more glucose uptake could be observed in FDG/PET scans of adipose depots from patients that perceived to be or felt cold (132). Moreover, apparent hypermetabolic human BAT could even be reversed in patients with a warming protocol, suggesting the possibility of cold temperature-induced human thermogenic fat cells (133). These findings were solidified in a series of FDG/PET-scans, immunohistochemical, and genetic studies identifying, unequivocally, the presence of adult human thermogenic fat cells at distinct anatomical positions (cervical, supraclavicular, axillary, mediastinal, paraspinal, and abdominal) (128–131). Interestingly, a study using single-photon emission computed tomography (SPECT/CT) coupled with lipid tracers identified new mouse BAT depots having a similar topology to human adipose deposits (134). Further histological and molecular characterization of these putative human BAT depots will aid in defining their thermogenic competencies and metabolic capacity.

The discovery of human BAT spurred multiple studies examining the role of thermogenic fat in human health, which showed a negative association with body mass index and adiposity, heralding thermogenic fat as an anti-obesity target (135). The presence of thermogenic fat is also positively correlated with insulin sensitivity (136). Moreover, higher human BAT prevalence is associated with a reduction in cardiometabolic diseases such as hypertension, coronary artery disease, and congestive heart failure (137). Human BAT presence is also correlated with body fat redistribution, protecting metabolically healthy subcutaneous fat while reducing visceral adiposity. These changes were also associated with fewer incidents of type 2 diabetes and fatty liver disease (138). Metabolically, PET scanning, using both glucose and lipid tracers, has successfully demonstrated the capacity of BAT to consume substrates and create a metabolic sink (139). For example, using the fatty acid tracer, ^18^F-fluoro- thiaheptadecanoic acid (18FTHA) to quantify BAT oxidation in cold-exposed healthy males, showed a substantial increase in nonesterified free fatty acid uptake (140). Correspondingly, BAT radio-density increased after cold exposure suggesting that brown adipocytes were actively lipolytic. Indeed, BAT activation in humans is associated with accelerated lipid metabolism and a 45-fold change in BAT mitochondrial thermogenic potential (139). Furthermore, BAT-induced glucose and free fatty acid uptake are inversely correlated with shivering and associated with a 1.8-fold increase in total energy expenditure in cold-exposed humans (140). Interestingly, in mice lacking Ucp1, FDG/PET scans reveal similar glucose uptake, suggesting fuel uptake can be separated from its oxidation (141). Therefore, uptake and oxidation must be consideration when probing human thermogenic fat function.

How much thermogenic fat do humans have? This question is challenging to answer due to whole-body imaging techniques—PET/MRI—to identify and define anatomical BAT locations (142). A further confounder is that BAT amount and presence vary by age and sex (143). More troubling is that BAT activity and amount are inversely associated with body mass index, with obese patients tending to have low to undetectable levels of BAT. In addition, the amount of BAT present in lean young adults’ ranges from 0.1 to 0.5% of total body mass (144). What’s more, humans, proportionally, have significantly less BAT than smaller mammals, which appears insufficient to support metabolic demand. In support, Wolfrum and colleagues used single nuclei RNA sequencing to profile mouse and human adipocyte heterogeneity to identify a subpopulation of high temperature (warming) induced adipocytes (145). This subpopulation appears to negatively influence the adipose tissue niche by modulating acetate bioavailability, stifling the number of thermogenic fat cells and activation. Human biopsies revealed an abundance of these anti-thermogenic adipocytes, which may explain why human fat has a lower thermogenic potential (145). Nevertheless, it remains to be determined if or how these cells function to control thermogenic fat bioavailability and their potential role in systemic metabolism and obesogenic conditions. While humans have metabolically activated BAT, which could influence energy expenditure, the critical question is: can humans recruit enough BAT to offset obesity and metabolic disease? Enthusiastically, both retrospective and prospective studies show that even limited thermogenic tissue can be inversely correlated with body mass, suggesting metabolic utility (146, 147).

### To beige or to brown

At the cellular level, brown and beige adipocytes appear morphologically similar. Moreover, isolated mouse brown and beige adipocytes have the equivalent thermogenic capacity and Ucp1 induction after norepinephrine administration (148). Additionally, a combination of MRI scans and molecular analysis revealed that human infant interscapular BAT resembles classical mouse interscapular BAT. In contrast, human supraclavicular and retroperitoneal depots genetically mirrored rodent beige adipose tissue (149). To test if there are unique differences between beige and brown adipocytes, Wu and colleagues evaluated global gene expression profiles and identified common thermogenic genes but also found divergent genes within beige adipocytes, such as Tmem26 and CD137. Moreover, human thermogenic fat has a genetic signature resembling rodent beige adipocytes rather than classical brown adipocytes (150–152). Yet this comparison could be masked due to the physiological state of the mice. For example, standard mouse procedures are performed at room temperature (~20-23°C); however, this is considered a thermal stress for mice, resulting in metabolic rate doubling (91). This would be equivalent to constant exposure of an unclothed human at 10°C (153). Humans have near domination over their thermoneutrality by modifying their environment and mitigating temperature fluctuations (i.e., clothing). Thus, is the correct comparison between thermoneutral humans and thermal stressed mice or thermoneutral humans and humanized mice? This debate is ongoing, and future research to elucidate the cellular identity of human thermogenic fat will be critical for understanding the molecular underpinning and focusing therapies (154).

While multiple studies have shown that cold exposure can stimulate human thermogenic fat, can pharmacological approaches be considered? Targeting Adrbs in most circumstances appears contraindicative for thermogenic intervention due to adverse cardiac events. However, a specific Adrb3-agonist, Mirabegron, has been designed and food and drug administration (FDA)-approved to treat patients with overactive bladder (155). Using a single dose (50 mg), Mirabegron increased glucose uptake and energy expenditure in healthy adult humans (156). Unfortunately, but as possibly anticipated, adverse cardiac-associated events (i.e., increased blood pressure) were observed in Mirabegron-treated patients (156, 157). Yet, treating obese subjects with mirabegron activated thermogenic fat tissue and increased glucose uptake and was associated with few cardiovascular events (158). While cold exposure and Adrb3-agonist stimulate thermogenesis, these methods are inconvenient, cumbersome, and elevate heart rate and blood pressure. Going forward, developing pharmaceuticals diverging from Adrb activators and focusing on alternative thermogenic pathways or stimulating fat cell progenitors will be critical. However, these endeavors may prove challenging as thermogenic targets could have opposing effects on systemic physiology.

## The decline in thermogenic fat

**Figure 3:**
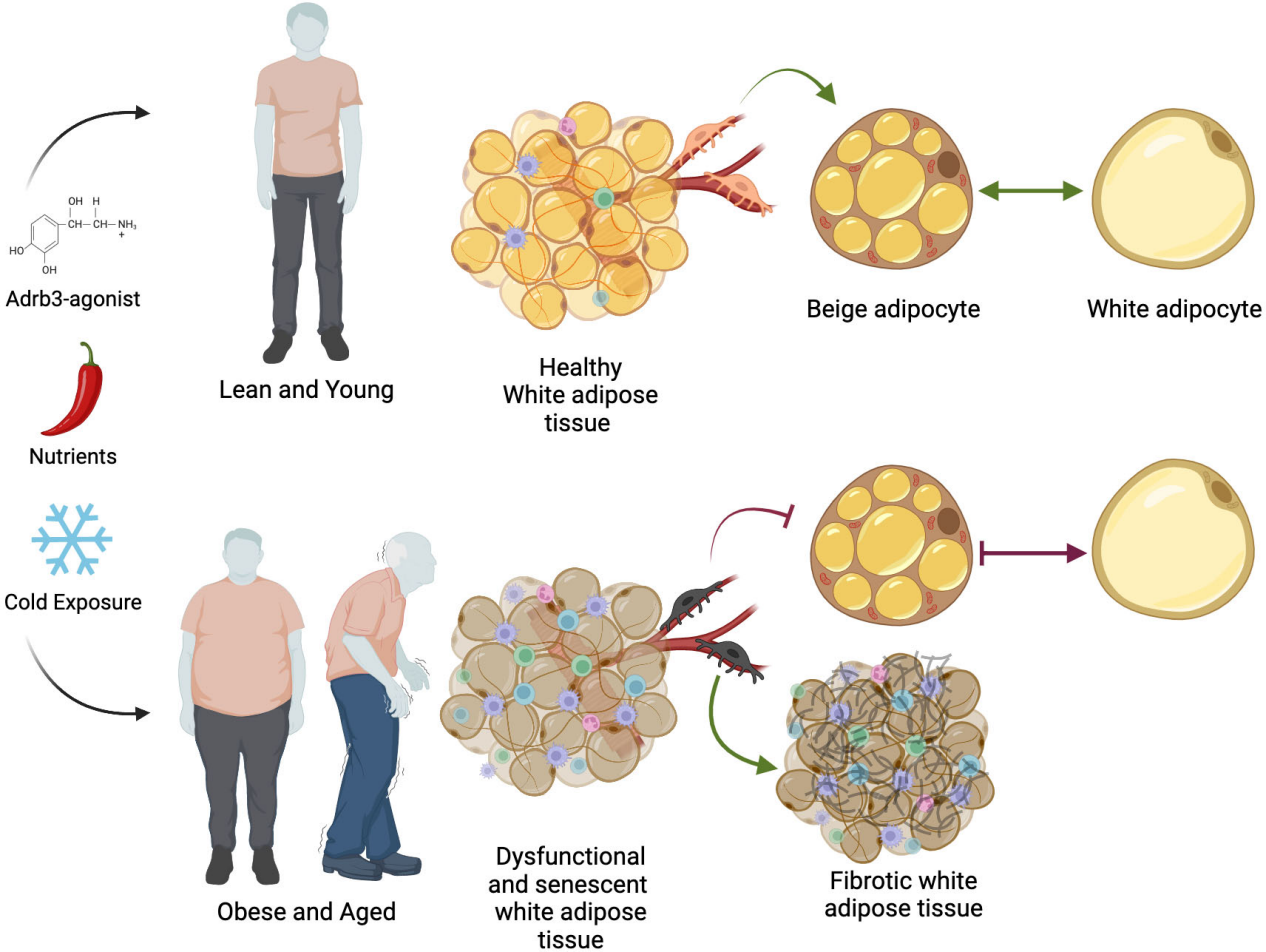
The decline in thermogenic fat biogenesis. Environmental, biochemical, and dietary activators can induce beige adipocyte formation in humans. In lean and young individuals, healthy white adipose tissue is freely able to perform beige-white adipocyte conversion depending on stimuli. In obese and aged settings, beige APCs become disrupted and display a senescent signature, becoming unresponsive to beige adipogenic stimuli. Moreover, changes in beige APCs facilitate fibrotic tissue replacement and inflammation. Additionally, aging is associated with changes in mitochondria function and thyroid hormone sensitivity resulting dysregulated white-to-beige interconversion. Together, age- and obese-associated changes in adipose tissue homeostasis stifle thermogenic fat biogenesis and metabolic protection. Illustration created with BioRender.com.

Thermogenic fat has clinical utility to counter metabolic imbalance and reduce adiposity; however, a key challenge has been the effectiveness of inducing beige fat in aging and obese humans. While short-term cold exposure increases insulin sensitivity and glucose uptake in healthy adults with active thermogenic fat (136), this effect is significantly blunted in obese subjects compared to normal-weight individuals (159). For reasons not entirely understood, the ability to generate cold-induced beige fat declines with increased adiposity and age (**Figure 3**). For example, Yoneshiro et al., showed that less than 10% of BAT activity could be detected in 50- and 60-year-old men and women, whereas young adults (~20s) showed >50% of BAT activity after 2 hours of cold exposure (160, 161). Similarly, in mice, aging causes a programmed decline in beige fat development within subcutaneous adipose tissue (162). These observations could, in part, contribute to the age-dependent accumulation of fat mass and the so-called “slowing” of metabolism in aging mammals. What is clear is that aging is associated with changes in visceral adiposity, reduced exercise capacity, impaired muscle maintenance and growth, increased peripheral nerve damage (neuropathy), the inability to regulate body temperature, and the constant feeling of cold (163, 164). Consistent with dysregulated homothermic control is the decrease in sympathetic innervation in aging humans (165). In agreement, while basal adipocyte lipolytic rates remain similar between young and aged human subcutaneous fat depots, catecholamine-stimulated lipolysis is 50% less (166). The disruption to transmit catecholamine signaling appeared to depend on hormone sensitive lipase activation and not necessarily, Adrb expression (166). In contrast, rodent studies suggest that sympathetic tone is elevated in aged mice demonstrating, more sympathetic stimulation (167). However, aging does not affect Adrb3 density on brown adipocyte membranes, suggesting that stimulation is preserved but not activated (168). Notably, few studies have examined the relationship between BAT activity and human genetic variation. For instance, single nucleotide polymorphisms found in the human UCP1 and ADRB3 genes are correlated with the age-dependent decline in thermogenic fat and augmentation of adiposity in healthy Japanese adults (169, 170). Nevertheless, what might account for the overall decline in thermogenic fat biogenesis and activation in the aging population?

### Senescence

Age-associated changes in beige fat biogenesis also appear to be driven, in part, by beige progenitor dysfunction, ultimately becoming unresponsive to cold signals. The reason(s) why these beige APCs become dysfunctional is vague, but it may emanate from a cellular aging-senescent-like phenotype. That is, with age, beige APCs begin to express a bevy of senescent markers (p16^Ink4a^ and p21), senescence activated β-galactosidase, and the senescence-associated secretory phenotype (SASP) (171). Age-associated changes in SASP signals, such as Tnfα and IL6, can further disrupt the tissue microenvironment facilitating inflammatory, necrotic, and senescent signals onto neighboring cells (172, 173). Consistent with the idea that senescent cells can be detrimental to tissue function, elegant studies designed to remove senescent cells—genetically or pharmacologically—from adipose tissue have demonstrated significant improvement in adipocyte tissue function and health (174). For example, removing white adipose tissue resident p21^high^ cells prevents and alleviates insulin resistance in obese mice (175). In agreement with these findings, deleting senescence inducers reinstates beige APC adipogenic potential and some metabolic benefits in aged mice. Strikingly, these age-dependent changes in senescence machinery and beige adipogenic failure were confirmed in white adipose tissue stromal cells from adolescent and adult humans (171). Mechanistically, cellular aging signals involve the activation of the p38/MAPK pathway, acting as a central node in regulating cellular senescence and aging genes. In support, upregulating the p38/MAPK senescence pathway within beige APCs prematurely blocks beige adipocyte development in young mice. Alternatively, pharmacologically blocking p38/MAPK in aged beige APCs rejuvenated beige fat development and improved aspects of systemic metabolism (171). Furthermore, p53, acting downstream of p38/MAPK signaling, is upregulated in aged white adipose tissue, and genetic and pharmacological blockade of p53 restores beige adipogenesis, potentially *via* mitophagy (176) (**Figure 3**). Yet, several important questions remain regarding the cellular aging processes; for example, what are the upstream signaling events that promote p38/MAPK hyperphosphorylation? For that matter, how does white adipose tissue aging affect the beige APC niche and vice versa? Answers to these questions might provide preventive clues for new senolytic-like compounds to restore beige fat in aged humans to boost metabolism and prevent middle-aged waist expansion (174, 177, 178).

While cellular aging appears to contribute to age-associated beige adipogenic failure, other cellular aging mechanisms that facilitate thermogenic fat decline may exist. For instance, Seale and colleagues demonstrated that cold exposure prevented beige APCs from adopting a fibrogenic phenotype (**Figure 3**) (179). In contrast, aging appeared to promote the fibrogenic phenotype, which correlated with the loss of Prdm16. Indeed, restoring Prdm16 levels in aged mice resulted in the rejuvenation of beige adipogenesis. This Prdm16-mediated process appears to involve β-hydroxybutyrate, a ketone body, which may reflect why ketogenic diets may successfully reduce adiposity and improve systemic metabolism.

### Mitochondrial dysfunction

Age-associated changes in mitochondria biogenesis and function may also facilitate the decline in thermogenic fat development. Mitochondria dysfunction is characterized by increased DNA damage, elevated reactive oxygen species, and decreased mitochondria biogenesis and oxidative phosphorylation (180). Thermogenic fat cells represent a unique cell type to examine mitochondrial biogenesis and clearance due to increased mitochondria number and activity. Damaged mitochondria are removed by a process known as mitophagy, an autophagic process targeting mitochondria (181). Because thermogenic fat cells can interconvert into white adipocytes, mitophagy is engaged to clear mitochondria, expediting the conversion. For instance, Kajimura and colleagues demonstrated that mitophagy removes the excess mitochondria to adopt a white fat–like phenotype after the withdrawal of cold-stimulus or Adrb3-agonist (182). Pharmacologically blocking or deleting genes associated with autophagy-mediated mitochondrial clearance preserves the beige adipocyte phenotype and function. Moreover, blocking mitophagy promotes the perdurance of beige fat and prevents obesity and glucose intolerance (183). Further investigation into age-associated decline in mitophagy could trigger the precipitous decline in thermogenic fat. For instance, aging may be associated with changes in autophagy-related genes, such as ATG5, ATG12 or PARK2, rapidly converting thermogenic fat cells into white adipocytes (182, 183). Consistent with this notion, the age-related downregulation of the epigenetic eraser, Lsd1, a genetic regulator of the mitophagy machinery, leads to the inability to induce thermogenic fat. Alternatively, overexpressing Lsd1 promotes a thermogenic fat cell phenotype (184, 185). Moreover, a recent report indicated that brown adipocytes release extracellular vesicles that contain oxidatively damaged mitochondrial parts to avoid thermogenic failure. BAT resident macrophages remove these extracellular vesicles; however, thermogenesis is dampened if macrophage function or accrual is disrupted (186). Further investigation into mitophagy and mitochondrial regulatory processes could provide new insight into the regulation of thermogenic fat cell viability and longevity, especially in the aged setting.

### Thyroid hormone

Thyroid hormones, triiodothyronine (T3) and thyroxine (T4), which can activate the thyroid hormone receptor to control gene transcription, can regulate BAT activation and differentiation thereby governing energy expenditure (187–190). While hyperthyroidism causes hyperthermia, the mechanisms remain controversial but may be partly mediated by skeletal muscle and by elevating the body’s temperature setpoint (191). This effect is exemplified in rodents demonstrating that T3 augments heat loss *via* the tail vein (192). Yet, T3 can also directly stimulate hypothalamic neurons to engage BAT thermogenesis (193, 194). While T3 and T4 can induce Ucp1 expression and thermogenic fat formation, aging is associated with a decrease in serum T3 and a reduced conversion of T4 to T3 (195). Correspondingly, human association studies suggest that UCP1 expression corresponds with circulating thyroxine serum levels, suggesting that lower thyroid hormone status could indicate reduced thermogenic potential, particularly with advanced aging (190). Yet, thyroid hormone-induced Ucp1-positive beige adipocytes may be decoys. Intriguingly, while these cells resemble beige adipocytes and express Ucp1, they appear metabolically inactive without adrenergic stimulation (196). Moreover, selective hyperthyroidism in Ucp1-null mice maintains metabolic and thermogenic responses (197). Suggestively, thyroid-induced hyperthermia is independent of brown and beige adipocytes and may involve Ucp1-independent thermogenic thyroid hormone mechanisms (196). A longitudinal study examining thyroid carcinoma patients undergoing thyroidectomy that receive the thyroid hormone (T4) replacement analog, levothyroxine, displays a boost in energy expenditure and glucose uptake into thermogenic fat depots (198). Consistently, a T3 analog, liothyronine, significantly decreased insulin resistance in patients with an insulin receptor mutation (199). While the data suggest that thyroid hormone and its analogs can stimulate hyperthermia, it may be unlikely that these effects are solely thermogenic fat dependent. Moreover, it is unclear how thyroid levels and thyroid receptor signaling mediate thermogenic fat biogenesis in aged mammals. Further, thyroid analogs show promise in thyroid cancer clinical trials, yet can these thyroid mimetics stimulate active thermogenic fat within the obese and aging populations to augment energy expenditure, remains to be clarified.

### Immune cells

Obesity and aging are associated with increased pro-inflammatory cytokine production in white and brown adipose tissue (200). These changes in cytokine composition further recruit and activate M1 macrophages and several other immune cell populations that facilitate tissue disruption and chronic low-grade inflammation (**Figure 3**). Moreover, pro-inflammatory cytokines disrupt cold-induced thermogenesis by directly suppressing thermogenic gene expression and dampening metabolic fluxes. Cold temperature exposure can remodel white adipose tissue immune cell composition, including M2 macrophages, mast cells, eosinophils, and type 2 innate lymphoid cells (ILC2s). For example, while ILC2s can support tissue homeostasis, they appear necessary for mediating energy balance in response to changes in environmental temperatures (201, 202). ILC2s are recruited and activated by the cytokine, interleukin-33 (IL-33), and once activated, produce an opioid-like peptide, methionine-enkephalin (MetEnk) peptide, that can stimulate beige fat development. It is unspecified if MetEnk mediates *de novo* beige adipogenesis or white-to-beige interconversion or its function mechanism (201). Alternatively, ILC2 and eosinophil byproducts, such as IL-13 and IL-4, have been shown to directly act on beige adipocyte progenitors to stimulate proliferation and differentiation (202, 203). Likewise, beige APCs also have been shown to produce and secrete IL-33 to facilitate a positive-beige-ILC2 adipogenic feedback circuit (204, 205). Nevertheless, this circuit may deteriorate with advanced aging and obesogenic signals, causing changes to beige fat generation, yet the exact mechanisms remain unknown (206). Moreover, a recent study showed the emergence of an age-dependent regulatory cell (ARC) population within white adipose tissue. ARCs resemble APCs but lack adipogenic capacity; instead, these cells secrete high levels of pro-inflammatory chemokines, including Ccl6, to inhibit the proliferation and differentiation of neighboring adipose precursors (207). Yet, the involvement of ARCs in beige fat biogenesis is unknown. Consistent with changes in pro-inflammatory cytokines, suppressing interleukin-10 (IL-10) induces thermogenic gene expression, whereas deleting the IL-10 receptor enhances beige fat formation and blunts obesity (208). More broadly, using single-cell RNA sequencing, Farmer and colleagues developed a comprehensive atlas of the cellular and transcriptional changes varying between cold temperature exposure and Adrb3-agonism. The analysis revealed that Adrb3 stimulated the interferon/Stat1 pathways, favoring myeloid immune cell accrual, whereas cold promoted lymphoid immune cell recruitment (209). Yet, the physiological function and molecular mechanisms of why certain beige stimuli favor specific immune populations remain to be fully understood.

## The effect of exercise on thermogenic fat

While the beneficial effects of exercise on human health are well appreciated, the ability of exercise to induce thermogenic fat biogenesis and activation appears mixed and may be species-specific (reviewed in (210)). For instance, a bout of exercise in rodents can generate thermogenic fat cells and upregulate the expression of thermogenic genes such as Prdm16 and Ucp1 in white adipose tissue (211, 212). Yet, in humans, there is a lack of association between exercise and thermogenic fat production in white fat depots (213–216). For example, lean and obese individuals that underwent endurance exercise training for 10-16 weeks did not elevate thermogenic gene expression in subcutaneous adipose tissue (217). Additionally, no changes in Ucp1 expression have been observed in human populations with active lifestyles compared to sedentary populations (218, 219). It is still being determined why these differences between humans and rodents exist. As discussed above, a potential confound could be the differences in ambient temperatures in which the experiments were conducted. These findings also pose an interesting physiological question, why would exercise generate thermogenic fat cells? The rodent exercise-induced beiging appears counterintuitive to the actual heat dissipation function of thermogenic fat cells (220, 221). A possible explanation is that exercise could diminish BAT function to maintain homeostatic temperature. This is conceivable because both thermogenic fat and exercise increase energy expenditure and thermogenesis (220, 221). Simplistically, exercise can also stimulate sympathetic activity, thus, by default, enhancing thermogenic fat biogenesis (222). On the other hand, exercise can stimulate the release of muscle- and adipocyte-derived secretory hormones (myokines and adipokines) that can facilitate thermogenic fat development. For example, irisin (223), myostatin (224), meteorin-like1 (Metrnl) (225), lactate (226), and b-aminoisobutyric acid (BAIBA) (227) can be released from muscle during exercise to stimulate thermogenic fat development. Even less convincing is the effect of exercise on classical BAT. For example, several studies have reported no effect or a decrease in BAT activity in response to exercise (212, 228). However, cold water swim tests suggest cooperation between cold temperature exposure and exercise to increase BAT mass. Nevertheless, human BAT exercise studies are lacking, which could be attributed to the decline in thermogenic fat tissue in aging humans. Additional exploration into the physiology and molecular mechanisms underlining the effects of exercise on thermogenic fat adaptation and decline remains to be fully elucidated.

## The effect of diet and nutrients on thermogenic fat

Most individuals do not prefer to spend several hours per day in a cold chamber activating thermogenic fat. Thus, developing and implementing more tolerable and potentially desirable methods should be employed to promote thermogenic fat biogenesis. For thousands of years, diet, nutritional ingredients, and natural compounds have provided therapeutic and medicinal effects on human metabolism and whole-body physiology. These alternative approaches could harbor tactics to avoid cold exposure or synthetic compounds altogether. For instance, besides cold exposure, the SNS can also be engaged by stress, inflammation, and diet. Diet is particularly interesting because it can be a modifiable and controllable aspect of human life. Furthermore, diet appears to induce obligatory and facultative thermogenesis. Obligatory thermogenesis entails heat generation during digestion, absorption, and processing of dietary components, often referred to as the thermogenic effect of food. Facultative thermogenesis is the breakdown of macronutrients and the activation of classical BAT thermogenesis, resulting in heat dispersion from food energy. Yet, the role of diet in controlling thermogenesis has not been largely undefined; however, studies using specific diets, such as the ketogenic diet, suggest that certain food may potentiate or even block thermogenic fat activity (229). Moreover, single-meal studies suggest that food can alter BAT activity immediately. For instance, FDG PET scanning revealed less glucose uptake into BAT depots in healthy human participants 90 minutes after a meal (230, 231). These results suggest that diet may negatively impact BAT activity, yet a possible explanation could involve insulin mediated FDG uptake into muscle, thereby limiting the free FDG for BAT utilization. This explanation could justify the underestimate in BAT glucose uptake—alternative measurements, such as oxygen uptake, may be more applicable for measuring mitochondrial function and BAT activity (232). Indeed, a recent report demonstrated that BAT oxygen and blood flow rose immediately after a single meal. In further support, participants with more BAT had higher total energy expenditure after meals than individuals with lower BAT activity (233). These studies suggest that diet can affect BAT activity; however, more investigation is needed to elucidate how cold exposure and diet could be coupled to control BAT activity.

In addition to diet and meals, nutrient composition of food may also affect thermogenic fat biogenesis and activity. For example, capsaicin is a pungent compound found in hot peppers that can induce hypermetabolism and sweating upon consumption (234). Multiple studies using rodents and humans have shown the beneficial effects of acute and chronic administration of capsaicin on total energy expenditure and fatty acid oxidation (234–238). Furthermore, overweight or obese subjects that ingested red peppers with meals had reduced adiposity, increased fatty acid oxidation, and enhanced whole-body energy expenditure (239). Nevertheless, due to its spiciness, capsaicin is less palatable, preventing ingestion of large quantities, thus, making capsaicin less desirable and to a lesser extent, a convincing beige fat therapy (240). However, capsinoids, analogs of capsaicin but without the pungency, can be as potent as capsaicin in boosting energy expenditure in rodents and humans (241). For example, after two hours of cold exposure (19°C), test subjects that had received capsinoids (9 mg) increased FDG uptake into adipose depots within the supraclavicular and paraspinal regions. Moreover, energy expenditure increased three-fold in response to cold exposure after oral ingestion of capsinoids but not under warm temperature conditions (242). Mechanistically, capsinoids are a class of vanilloid compounds that directly bind to the TRPV1 channel to increase intracellular calcium signaling, thereby increasing thermogenic action, potentially *via* Ucp1 and mitochondrial biogenesis (98, 243). Notably, it appears that two capsinoid molecules may be needed to activate the channel (244). In addition, mice lacking TRPV1 are completely blocked from capsaicin’s effect on energetics and beige fat formation (245). Like capsinoids, other naturally occurring compounds such as allicin and alliin from garlic and onion and ginger-derived compounds can activate TRPV1, suggesting a broad spectrum of nutritional compounds that could elicit thermogenic responses (246, 247). Thus, while capsinoids provide unique insight into thermogenic activation *via* non-adrenergic methods, other potential pungent and non-pungent compounds could be exploited as thermogenic ingredients (248). For example, menthol, a compound found in mint, has been shown to stimulate thermogenesis and increase energy expenditure in rodents *via* TRP channel activation (249). In addition, other food molecules and nutrients, such as retinoic acid, resveratrol, and fish oils, have been shown to stimulate thermogenic responses to improve metabolic fitness (250–252). For example, treating obese mice with slow-release retinoic acid pellets reduced body weight and fat mass, improved glucose clearance, and upregulated a thermogenic program *via* two nuclear hormone receptors—retinoic acid receptor (RAR) and Pparβ/δ[252]. However, the functional utility of retinoic acid and other natural compounds in humans has yet to be fully determined (252, 253). Additionally, future investigation into relevant mechanisms of nutritional components will highlight the efficacy of these natural compounds in augmenting thermogenic fat formation. Moreover, might these natural ingredients have the potential to avoid age and obesity-induced effects on thermogenic function? Promisingly, the convenience of natural ingredients/compounds could be a strategy for obesity prevention by combining them with dietary foods, such as the marketing success of spicy chocolate.

## Conclusion and prospective

In the United States, the adult obesity prevalence is 41.9%, but even more staggering is the 19.1% obesity occurrence among children (2-19 yrs.) (254). The defining feature of obesity is excess white fat mass, increasing the risk for metabolic disorders and premature death (255). Thus, identifying potential and tangible therapies is rapidly needed to counteract this public health problem (254). The ability to generate thermogenic fat is a feasible and highly desirable anti-obesity target due to its ability to increase energy expenditure and futilely burn substrates (**Figure 1**) (4, 125, 255). Unequivocally, the generation of thermogenic fat in rodents and healthy humans suggests metabolic potential (**Figure 1**) (256–258). Skeptically, it could and has been argued that the amount of thermogenic fat needed to alter whole-body human metabolism is more than what can be generated. Still, the human thermogenic fat field is nascent and has only begun detecting human thermogenic fat with the possibility of new depots being discovered. The development and advancement of imaging and the need for substrate tracer technology are considered critical for defining and characterizing thermogenic fat deposits and metabolic sink potential. Moreover, studies involving obese subjects will be required to understand human thermogenic fat-induced metabolic reprogramming. Additionally, cold exposure may provide its own therapeutic effect, independent of thermogenic fat development, which may boost metabolism, lower adiposity, and reduce inflammation.

Several rodent therapeutic strategies have successfully targeted thermogenic fat; however, these tools may not apply to human adipocytes. For instance, a recent study showed that Adrb2 drives human thermogenic fat biogenesis, not Adrb3 (259). But another study suggests that Adrb3 does mediate human thermogenic fat biogenesis (260). Nevertheless, as observed from Adrb agonists, such as Mirabegron, targeting Adrbs can alter energy homeostasis but may be contraindicative in the larger population. Therefore, the development of targeted therapies, such as gene-based or tissue-specific pharmaceuticals, is ideal, providing precision in thermogenic fat biogenesis and limiting off-target side effects (261). Furthermore, thermogenic adipocytes appear heterogenic and are derived from various cellular sources. Hence, a continued examination of cell ontology, lineage tracing, and genetic profiling will help unearth the complexity and lineage relationships. For example, while single-cell transcriptomics provides resolution on cellular populations and their trajectories, incorporating lineage tracing and spatial-niche reconstruction technology such as RNA-fluorescence *in situ* hybridization (FISH) will provide information on cellular utility, tissue microenvironment, and adipogenic competency (262, 263). Previous studies have posited that adipose tissues are homogenous; however, identifying brown and beige adipocyte subpopulations provides new insights into how adipocytes are potentially formed, activated, and metabolically induced.

A potential, but unexplored node of nutritional research, is the advancement of dietary thermogenic inducers (248). Critically, mechanistic insights into nutritional thermogenic regulators still need to be improved, which would be necessary to provide tailored thermogenic fat development. For instance, do capsaicinoids stimulate thermogenic progenitors or white-beige interconversion, and what is the metabolic substrate preference of capsaicinoid-induced thermogenic fat? While these questions may appear nuanced, they could direct whether capsaicinoid supplements or other nutrient thermogenic activators will be effective depending on the patient’s metabolic phenotype. Moreover, these new thermogenic regulators could offer a vast pharmacopeia of agents to ignite thermogenesis, hoping to circumvent pesky cold exposure altogether. However, these efforts may all be in vain, especially if thermogenic fat fails with age, beginning in our mid-30s (160). For that matter, increased adiposity also slows the development of thermogenic fat biogenesis. Thus, how can thermogenic fat be renewed in the aging-obese population? Studies targeting the hallmarks of age-dependent thermogenic fat failure will be vital for thermogenic fat renewal. Moreover, aging is a process throughout life, not a terminal age, which may be a critical point for intercepting its decline (264). Nevertheless, there may be hope with the development of senolytics and other pharmaceuticals that have shown promise in restoring thermogenic fat in obese or aged subjects. In conclusion, the advancement in thermogenic fat formation over the past decade has been substantial. Yet, developing new biomedical technologies coupled with basic cellular and molecular biological research will foster new developments into the full potential of thermogenic fat as a possible therapy to thwart obesity and metabolic disease.

## Author contributions

SX wrote the draft and revised the review. SX, DL, and DB revised and wrote the final version. All authors contributed to the article and approved the submitted version.
